# Low-dose isoflavone aglycone alleviates psychological symptoms of menopause in Japanese women: a randomized, double-blind, placebo-controlled study

**DOI:** 10.1007/s00404-015-3849-0

**Published:** 2015-08-21

**Authors:** Asuka Hirose, Masakazu Terauchi, Mihoko Akiyoshi, Yoko Owa, Kiyoko Kato, Toshiro Kubota

**Affiliations:** Department of Obstetrics and Gynecology, Tokyo Medical and Dental University, Yushima 1-5-45, Bunkyo, Tokyo 113-8510 Japan; Department of Women’s Health, Tokyo Medical and Dental University, Yushima 1-5-45, Bunkyo, Tokyo 113-8510 Japan

**Keywords:** Flavonoids, Depression, Insomnia, Mood, Sleep

## Abstract

**Purpose:**

Many studies have demonstrated the effectiveness of isoflavones on menopausal symptoms; however, these mostly used high dosages. Because high-dose isoflavone may result in endometrial hyperplasia, we investigated whether low-dose isoflavone aglycone alleviates menopausal symptoms similarly to high dosages.

**Methods:**

We conducted a randomized, double-blind, placebo-controlled study in 90 healthy women aged 40–60 years who had at least one menopausal symptom on the Menopausal Symptom Scale (MSS). The participants were randomized to receive active tablets containing ultralow-dose (12.5 mg/day; *n* = 30) or low-dose (25 mg/day; *n* = 30) isoflavone aglycone, or placebo (*n* = 30) tablets, for 8 weeks. Their menopausal symptoms were evaluated using MSS, Hospital Anxiety and Depression Scale (HADS), and Athens Insomnia Scale (AIS) before, and 4 and 8 weeks after treatment.

**Results:**

Eighty-seven women (97 %) completed the 8-week treatment. In the low-dose group, significant improvement was observed from baseline, in the following parameters: (1) HADS-depression subscale score, (2) AIS score, (3) MSS-somatic symptom score after 4 and 8 weeks of treatment, and (4) MSS-vasomotor symptom score after 8 weeks of treatment. The changes in scores on HADS-depression subscale and AIS from baseline to 8 weeks were significantly higher in the low-dose group than in the placebo group.

**Conclusions:**

Low-dose (25 mg/day) isoflavone aglycone significantly alleviated symptoms of depression and insomnia in Japanese middle-aged women.

*Clinical Trial Registration* UMIN-CTR UMIN000011876.

## Introduction

The advent of an aging society is raising various health-care issues in developed countries. Health promotion in women at midlife has gained paramount importance. Menopausal symptoms, such as hot flushes, night sweats, vaginal dryness, anxiety, depression, and insomnia, are among the major factors compromising the quality of life of middle-aged women. Although menopausal hormone therapy (MHT) effectively alleviates vasomotor and other menopausal symptoms, some women are unwilling or have contraindications to receive MHT owing to the onset of adverse effects, such as venous thromboembolism, stroke, and breast cancer. These women often use dietary supplements, including soy isoflavones, for the relief of menopausal symptoms. In this context, previously, grape seed proanthocyanidin extract has shown to alleviate physical and psychological symptoms of menopause, while increasing muscle mass and reducing blood pressure in middle-aged women [1].

Isoflavones are plant-derived flavonoids that are particularly abundant in leguminous crops and mimic the actions of estrogen by binding to estrogen receptors (ERs) α and β, and thus isoflavones are referred to as phytoestrogens. Isoflavones are contained in soybean or soy foods in two distinct chemical forms, glucosides and aglycones, and the latter are absorbed faster and in greater amounts in the human intestine than glucosides [2].

A number of clinical studies have suggested that soy or soy isoflavone may alleviate menopausal symptoms, especially vasomotor symptoms [3–11], supposedly through its estrogen receptor agonist [3, 5, 12–16] and antioxidant activities [17]. However, some randomized controlled trials failed to demonstrate their effectiveness [18–22]. The discrepancy may be because of the differences in participant age, duration since menopause, symptom severity, symptom assessment system, dose of isoflavones, proportion of aglycones contained, bioavailability of aglycones and glucosides, and baseline dietary intake of isoflavones.

In 2004, a randomized, double-blind, placebo-controlled trial including 319 postmenopausal Italian women revealed that 5-year treatment with 150 mg/day of soy isoflavones significantly increased the occurrence of endometrial hyperplasia (3.4 vs. 0 %, *P* < 0.05) [16]. Alarmed by this report, the Japanese Food Safety Commission propounded, in 2006, that the safe upper limit of daily intake of isoflavone aglycones should be restricted to 70–75 mg, thus limiting the amount that can be taken from dietary supplements to 30 mg or less [23]. Considering that most of the aforementioned studies that showed the effectiveness of isoflavone aglycones on menopausal symptoms were conducted using higher doses than the recommended levels, we examined whether lower doses of soy isoflavone aglycone improve menopausal symptoms or not.

## Materials and methods

A randomized, double-blind, placebo-controlled study was conducted from June 2013 to December 2013 at the Menopause Clinic of the Tokyo Medical and Dental University. The study protocol was reviewed and approved by the Tokyo Medical and Dental University Review Board, and a written informed consent was obtained from all participants. The study was conducted in accordance with the Declaration of Helsinki.

Japanese women aged 40–60 years who had at least one menopausal symptom on the Menopausal Symptom Scale (MSS) were eligible for the study. Those who were already receiving MHT were excluded. The participants were classified as follows: premenopausal (regular menstrual cycles in the past 3 months), perimenopausal (a menstrual period within the past 12 months, but a missed period or irregular cycles in the past 3 months), postmenopausal (no menstrual period in the past 12 months), or had surgically induced menopause (hysterectomy) [24].

The participants were randomized into one of three groups to receive active tablets containing ultralow-dose (12.5 mg/day; *n* = 30) or low-dose (25 mg/day; *n* = 30) isoflavone aglycone, or placebo (*n* = 30). They were instructed to take the supplement for an 8-week period. The ultralow-dose and low-dose isoflavone aglycone, and placebo tablets, indistinguishable in shape, weight, and color, were manufactured and packaged by Kikkoman Corporation (Noda, Japan). Isoflavones contained in the tablet are all in aglycone forms: genistein, 51.8 %; daidzein, 43.3 %; and glycitein, 4.9 %. The women were instructed to take one tablet per day at any time of the day. Medication adherence was evaluated by collecting the packages of the supplements.

The participants sequentially numbered received supplement packages with the corresponding number assigned by the manufacturer. The content of each package, namely ultralow-dose or low-dose isoflavone aglycone, or placebo, was not revealed until the end of the study period, so that the allocation was concealed from both the participants and the investigators.

The menopausal symptoms of the participants were evaluated using the MSS, Hospital Anxiety and Depression Scale (HADS) and Athens Insomnia Scale (AIS) before and after 4 and 8 weeks of treatment.

The MSS has been used and validated in previous studies, in which patients rate the severity of ten menopausal symptoms at every visit [25]. The MSS evaluates vasomotor symptoms (hot flush, perspiration, and chilliness); somatic symptoms (irregular heartbeat, headache/dizziness, tiredness, and aching joints/muscles); and psychological symptoms (insomnia, irritability, and depressed mood) using a four-point Likert scale depending on how often each symptom affects their daily life: none (never, 0 point); mild (rarely, 1 point); moderate (sometimes, 2 points); or severe (very often, 3 points). Vasomotor and somatic symptom scores are calculated as the total score of three and four aforementioned symptoms, respectively.

Developed by Zigmond and Snaith as a questionnaire [26], HADS is a reliable instrument for screening clinically significant anxiety and depression in women visiting a general medical clinic, which has been translated into Japanese by Kitamura et al. [27]. The AIS was developed as a brief and easy-to-administer self-assessment questionnaire for determining the severity of insomnia defined according to the International Classification of Disease Tenth Revision. The internal consistency and test–retest reliability of AIS have been confirmed previously [28]. The current study was conducted in a manner similar to a previous one using grape seed proanthocyanidin extract, in which detailed information about HADS and AIS questionnaires is provided [1].

The body composition of the participants, including height, weight, body mass index, fat mass, and muscle mass, was assessed using a body composition analyzer (MC190-EM; Tanita, Tokyo, Japan). Their cardiovascular parameters, including systolic blood pressure, diastolic blood pressure, and heart rate, were also measured using a vascular screening system (VS-1000; Fukuda Denshi Co., Tokyo, Japan).

All statistical analyses were performed with GraphPad Prism version 5.02 (GraphPad Software Incorporated, CA, USA) using one-way analysis of variance, *χ*^2^ test, Kruskal–Wallis test, Wilcoxon signed-rank test, and Mann–Whitney test. *P* < 0.05 was considered to be statistically significant.

## Results

**Fig. 1:**
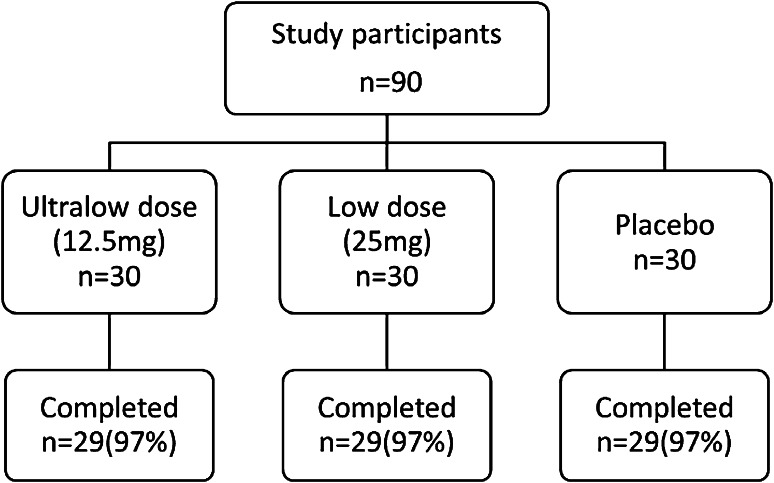
Participant disposition

A total of 90 middle-aged women were enrolled in the study and randomized to the ultralow-dose (*n* = 30), low-dose (*n* = 30), or placebo groups (*n* = 30); of these, 87 (97 %) completed the 8-week treatment period (Fig. 1). The baseline characteristics of the participants who completed the 8-week treatment are shown in Table 1. Their mean age was 48–51 years; 38–66 % of the women were premenopausal, while 10–17 % were perimenopausal, 21–41 % were postmenopausal, and 3–10 % had surgically induced menopause. No statistically significant differences were observed in the baseline characteristics among the study groups, except for the mean MSS-vasomotor symptom score: at baseline, the score in the ultralow-dose group was higher than in the other groups.

**Table 1:** Baseline characteristics

	Placebo (*n* = 29)	Ultralow dose (*n* = 29)	Low dose (*n* = 29)	*P*
Age and menopausal status				
Age, mean (SD), years	48.0 (5.7)	50.5 (4.7)	47.6 (4.9)	0.080^a^
Premenopausal/perimenopausal/postmenopausal/had surgically induced menopause, number (%)	15/5/6/3, (51.7/17.2/20.7/10.3)	11/5/12/1, (37.9/17.2/41.4/3.4)	19/3/6/1, (65.5/10.3/20.7/3.4)	0.290^b^
Physical symptom score, mean (SD)				
MSS-vasomotor symptom score	3.0 (2.2)	4.4 (2.1)	3.1 (2.2)	0.018^c^
MSS-somatic symptom score	4.0 (2.5)	4.7 (2.3)	5.0 (2.8)	0.446^c^
Psychological symptom score, mean (SD)				
HADS-anxiety subscale score	5.3 (2.5)	5.1 (2.3)	5.5 (3.3)	0.828^c^
HADS-depression subscale score	4.4 (2.3)	4.0 (2.1)	4.1 (2.1)	0.773^c^
Athens Insomnia Scale score	4.1 (3.0)	3.9 (2.6)	5.0 (3.3)	0.337^c^
Body composition, mean (SD)				
Height (cm)	158.1 (4.8)	157.4 (3.8)	158.6 (5.0)	0.596^a^
Weight (kg)	54.5 (7.7)	56.3 (9.0)	54.2 (7.7)	0.579^a^
Body mass index (kg/m^2^)	21.8 (2.7)	22.7 (3.2)	21.6 (3.2)	0.650^a^
Fat mass (kg)	14.9 (5.2)	17.0 (6.3)	14.7 (5.7)	0.279^a^
Muscle mass (kg)	37.3 (2.8)	37.1 (3.1)	37.2 (2.7)	0.972^a^
Cardiovascular parameters, mean (SD)				
Systolic blood pressure (mmHg)	117.3 (13.8)	117.2 (15.7)	121.0 (20.7)	0.651^a^
Diastolic blood pressure (mmHg)	63.7 (12.0)	67.8 (12.9)	67.6 (13.1)	0.406^a^
Heart rate (min^−1^)	69.7 (9.0)	73.3 (10.2)	69.0 (8.9)	0.188^a^

*SD* standard deviation, *MSS* Menopausal Symptom Scale, *HADS* Hospital Anxiety and Depression Scale

^a^One-way analysis of variance

^b^ *χ* ^2^ test

^c^Kruskal–Wallis test

**Fig. 2:**
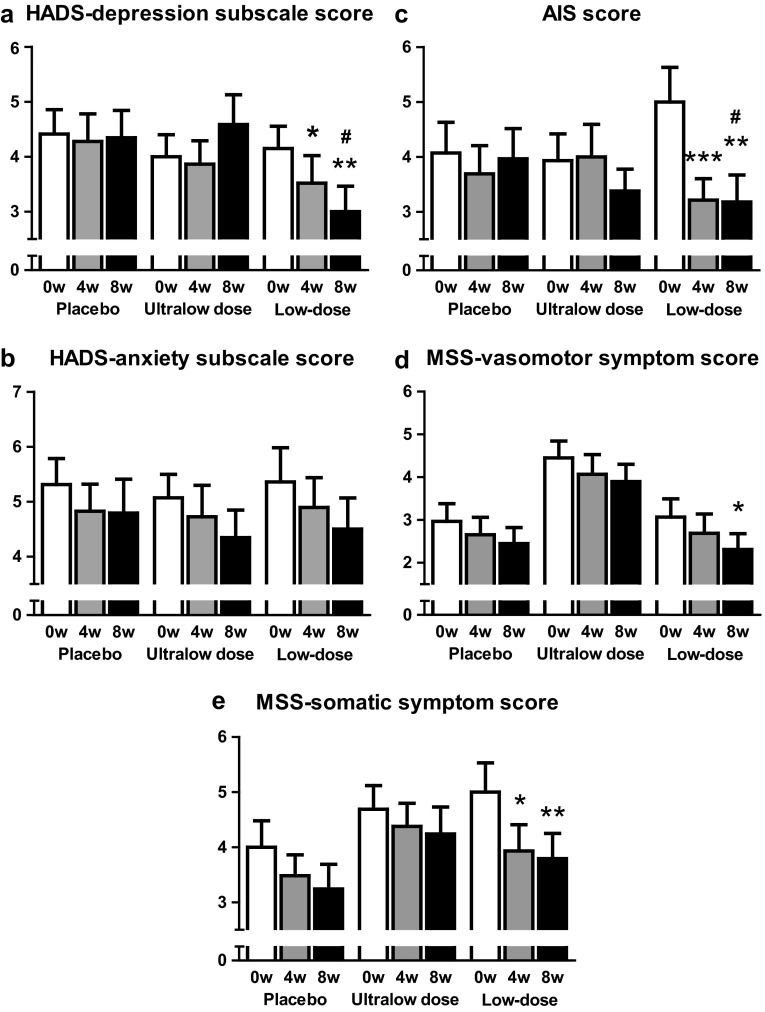
The changes from baseline in: **a** Hospital Anxiety and Depression Scale (HADS)-depression subscale score; **b** HADS-anxiety subscale score; **c** Athens Insomnia Scale (AIS) score; **d** Menopausal Symptom Scale (MSS)-vasomotor symptom score; and **e** MSS-somatic symptom score (mean and standard error, *P < 0.05, **P < 0.01, ***P < 0.001 versus baseline, Wilcoxon signed-rank test. ^#^ *P* < 0.05, change from baseline versus placebo, Mann–Whitney test)

We evaluated the severity of menopausal symptoms after 4 and 8 weeks of treatment in each study group. In psychological symptoms, the mean HADS-depression subscale score improved in the low-dose group after 4 and 8 weeks of treatment (4.1 ± 0.4 vs. 3.5 ± 0.5 and 3.0 ± 0.5, mean ± SEM, P = 0.032 and 0.003, vs. baseline) (Fig. 2a), but not in the ultralow-dose and placebo groups, whereas the mean HADS-anxiety subscale score did not change significantly in any of the groups (Fig. 2b). Similarly, the mean AIS score improved only in the low-dose group after 4 and 8 weeks (5.0 ± 0.6 vs. 3.2 ± 0.4 and 3.2 ± 0.5, *P* = 0.0006 and 0.002) (Fig. 2c). As for vasomotor and somatic symptoms, the mean MSS-vasomotor symptom score significantly improved in the low-dose group after 8 weeks (3.1 ± 0.4 vs. 2.3 ± 0.4, *P* = 0.010) (Fig. 2d), and the mean MSS-somatic symptom score significantly improved only in the low-dose group after 4 and 8 weeks (5.0 ± 0.5 vs. 3.9 ± 0.5 and 3.8 ± 0.5, *P* = 0.023 and 0.002) (Fig. 2e). There was no significant improvement in the ultralow-dose and placebo groups.

The changes in scores on HADS-depression subscale and AIS from baseline to 8 weeks were significantly higher in the low-dose group than in the placebo group: HADS-depression subscale score (−1.15 ± 0.34 vs. −0.07 ± 0.28, mean ± SEM, *P* = 0.033); AIS score (−1.82 ± 0.62 vs. −0.10 ± 0.38, *P* = 0.014).

Neither the body composition parameters nor the cardiovascular parameters changed significantly in any of the groups (data not shown).

During the whole study period, no treatment-emergent adverse event was reported from participants, even though laboratory tests or transvaginal ultrasound were not routinely performed.

## Discussion

We conducted a randomized, double-blind, placebo-controlled study to examine the effects of ultralow and low-dose isoflavone aglycone derived from soybeans on the symptoms of middle-aged women. As a result, the low-dose (25 mg/day) isoflavone aglycone significantly alleviated symptoms of depression and insomnia in Japanese middle-aged women.

Leguminous crops, utilized as foods from ancient times, especially in Asian countries, have been generally well tolerated without any remarkable health hazards. However, the alarm raised by the Italian study that showed an increase in endometrial hyperplasia after 5-year intake of 150 mg/day of isoflavones [16] prompted the Japanese Food Safety Commission to propound the safe upper limit of daily intake of isoflavone aglycones to 70–75 mg in 2006. This limitation reflected in the amount that can be consumed in dietary supplements, which was reduced to 30 mg or less [23]. Therefore, we conducted this study with ultralow-dose (12.5 mg/day) or low-dose (25 mg/day) isoflavone aglycone to examine whether lower doses of soy isoflavone aglycone could improve menopausal symptoms or not.

The most remarkable results in the present study were the significant improvements in psychological symptoms such as depression and insomnia with the use of the low-dose isoflavone. Compared with vasomotor symptoms, there are fewer randomized, double-blind, placebo-controlled studies that demonstrated the effects of isoflavones on psychological symptoms: two studies showed significant improvement in depression [29, 30] and one in insomnia [31]. Atteritano et al. showed improvements in depression with 54 mg isoflavone in a 2-year study, though the genistein and placebo tablets contained calcium carbonate and vitamin D as well [29]. Lipovac et al. revealed that 80 mg red clover-derived isoflavones administered for a 90-day period reduced depressive and anxiety symptoms [30], whereas Hachul et al. showed that 4-month treatment with 80 mg isoflavone was effective for insomnia [31]. However, all of these three trials used higher dosage and longer treatment periods than ours.

Although the mechanisms by which isoflavones improve depression and insomnia have not been fully explained, experiments in ovariectomized rats revealed that phytoestrogen genistein had antidepressant-like and anxiolytic-like effects probably through their estrogenic activities [32, 33]. Recent hypotheses suggest that estrogens affect the hippocampus, a limbic region implicated in mood disorders [34], through ER β [35]. Because isoflavones have higher affinity to ER β than to ER α [36], they may well be effective for psychological symptoms of menopause.

Psychological symptoms could derive from vasomotor symptoms, through so-called domino effect [37], with nightly vasomotor symptoms leading to insomnia, and then to depression, although some studies showed that depression and insomnia were independent of vasomotor symptoms [38, 39]. In the present study, we found that the changes in psychological symptoms were significantly higher in the low-dose group than in the placebo group, but not in vasomotor symptoms. Therefore, the domino effect would not likely be the underlying mechanism for the improvement of psychological symptoms. The effects of low-dose isoflavone on depression and insomnia were not significantly different by menopausal status either.

The reason why isoflavone at the low dose of 25 mg/day effectively alleviated menopausal symptoms in the current study may be because the soy isoflavones we administered were completely presented in the aglycone form, which is absorbed faster and in greater amounts than the glucoside form [2]. Six studies that administered isoflavones only in aglycone forms [3, 5, 6, 8, 10, 11] showed significant improvement in vasomotor symptoms. Alternatively, the soy isoflavone we used may have been effective because it was rich in genistein, which has higher estrogenic activity than daidzein and other phytoestrogens [40].

The present study has some limitations: (1) the number of participants was relatively small; (2) the study period was as short as 8 weeks; (3) the differences in symptom scores from baseline to the end of follow-up were relatively small, even though some of them reached statistical significance; and (4) the effects of isoflavones on endometrial thickness and histology were not evaluated. A study with a larger sample and longer duration, including women with more severe menopausal symptoms, could further clarify the effects of low-dose isoflavone aglycone on menopausal symptoms.

In conclusion, the present study revealed that low-dose (25 mg/day) isoflavone aglycone significantly alleviated symptoms of depression and insomnia in Japanese middle-aged women.
